# Effectivity–ecosphere–economics in nZEB retrofit procedures

**DOI:** 10.1007/s11356-018-2446-8

**Published:** 2018-06-23

**Authors:** Elżbieta Ryńska, Urszula Koźmińska, Joanna Rucińska

**Affiliations:** 1 Faculty of Architecture, Warsaw University of Technology, 55 Koszykowa Street, 00-659 Warsaw, Poland; 2 Faculty of Building Services, Hydro and Environmental Engineering, Warsaw University of Technology, 20 Nowowiejska Street, 00-653 Warsaw, Poland

**Keywords:** nZEB retrofit procedures in interdisciplinary design, Case study academic building, Energy efficiency, Health and well being

## Abstract

Sustainable development has by now become an element deeply integrated in the everyday design. It has many shades and may be found under many names. We speak about resiliency in design and procurement of passive, ecologic, plus energy, or nZEB buildings. Nevertheless, if we look closely, we may distinguish certain characteristic ideas. First, sustainable development of societies and urbanization processes should be consistent on a deeper level than presently, and be included within design processes, organization, and planning, as well as modernization and redevelopment procedures of existing urban tissue. Secondly, urbanization should be perceived holistically, as an interaction and harmonious development of both natural and manmade environments, with solutions based on the best technical and technological standards available. Lastly, described ideas are achievable only, if we include continuous cooperation between urban planners, architects, specialist consultants, as well as energy-efficient interdisciplinary solutions to achieve high standard energy measures. One of the thresholds is economic feasibility; the other is health and well-being of the users which should always be discussed as a priority. This paper—outside a brief theoretical approach to initial procedures in design management—will dwell on transformation and modernization of an existing building belonging to the Warsaw University of Technology, one of the oldest universities in Poland, its founding dating back to the beginning of the twentieth century. In 2015, a Nordic Finance Mechanism grant dedicated to the nZEB technology transfer from Norway to Poland was awarded to a group of researchers from Warsaw University of Technology and NTNU Trondheim. The main aim of the project is implementation of nZEB knowledge in Poland, as well as preparation of two integrated concept designs for public (University) buildings as exemplary case studies which could act as the benchmarks for other public buildings.

## Introduction

A lot has been said about building investment processes and design strategies, as well as environmentally friendly buildings and effective energy strategies. Many countries are far in the front line of advanced development, others are still struggling with the challenges which they are facing. Some already introduce technical definitions, system boundaries, and calculation methodologies, as well as set forth energy-based cost boundaries allowing achievement of nZEB requirements (Kurnitski et al. 2013). In other cases, it appears that cost optimal calculations set forth by the European methodology are not consistent with the level of national applications of the nZEB definitions including numerical indicators of primary energy expressed in kW/m^2^/a (Kurnitski et al. 2014). In fact, there is no official nZEB definition in Poland, as well as the limit drawn for the energy efficient expectancy appears to be very high—90 kW/m^2^/a. Additionally, representatives of the Scandinavian countries have noticed that there is little attention being paid to the relations between barriers in the decision-making process and challenges in the retrofitting process of nZEB renovations (Lindkvist et al. 2014). These inconsistencies may be the results of the large discrepancies in the current energy regulations found in various countries. Not only the performance levels are different, also the units in which the performance is measured differ. Primary energy, various energy frames, delivered energy, and CO_2_ emissions are used as the state indicators. These differences have impact on the building industry and complicate many processes including building design (Jagemar et al. 2011).

As part of energy efficient construction process, in the recent years, the nearly zero-energy buildings have received increased attention. The recast of Energy Performance of Buildings Directive—EPBD (EPBD Recast, Directive 2010/31/EU)—requires from 2019 that all new buildings occupied and owned by public authorities are nearly zero-energy buildings (nZEB), and by the end of 2020, all new buildings should also have the same status (Aelenei et al. 2015). When considering our urbanized space, it should be noted that most structures which have been built in the past 30 years under certain climatic conditions and according to the then—contemporary technical knowledge—will be present in our surroundings for at least next 30 years and will have to withstand adverse changing climatic impacts as well stricter effective energy expectations. This implies that most of our efforts should be placed on the modernization of the building substance constructed in various techniques and standards. Due to geographic distribution and diverse user expectations, as well as alternative approaches, it is evident that countries should develop individual strategies even if based on transformed knowledge and know-how of more developed countries (Christoforidis 2016).

Even brief research run on the possible alternative solutions used for modernization of the building substance proves that nZEB might be one of the major paths which should be followed. This choice may prove to be interesting in case of historic buildings subject to various constraints including architectural values. This challenge becomes even more important and concerns both the shell as well as the systems (Dalla Mora et al. 2015a, b; di Ruocco et al. 2016). Hence, the most important issue is that when designing according to nZEB strategies, all designers participating in the process have to understand the conditions of each particular development as well as use surrounding environmental parameters as complementary interactive influence on design solutions provided (Kantola and Saari 2016a, b). On the nZEB level, the parameters are usually discussed from the energy effective point of view and user comfort needs. In case of retrofit buildings, existing structures are even more diverse and any transfer procedure of existing solutions between countries is likely to be initiated with the achievement of new solutions before high-standard buildings will appear (Hamdy et al. 2013; Ó’Riain and Harrison 2016; Beccali et al. 2013; Dalla Mora et al. 2015a, b). The outcomes of the research impact the field of the nZEB design that contribute to the subjects also covered by other scientific researchers in view of energy and economic targets (Becchio et al. 2015), as well as identification and management of risks involved in the transition to nZEB (Kantola and Saari 2016a, b). It also dwells on the possible structure of the definition of nearly zero-energy buildings (Kurnitski et al. 2012). A lot of attention is focused on the interactions between architectural design and other disciplines, the choice and context awareness of such investments (Uribe et al. 2015). Some of the recently published papers, dedicated to the issue of decision-making and support tools, point out differences between countries (Kang 2015) and stress that each case should be carefully analyzed (Singh 2015).

The concept of net zero-energy building (nZEB), that is, the building in which the primary energy consumption is 0 kWh/(m^2^/a) is by now very widespread. Unfortunately, the approach is highly differentiated. In the case analysis, it is important to establish the definition of such a building, the indicators and technologies taken into account, and the individual national requirements. Such review of the most existing ZEB definitions and the various approaches towards possible ZEB calculation methodologies can be found in various papers (Marszal et al. 2011).

Described by Szalaya Z. and Zöldb A., approach for a single uniform definition of a net zero-energy building (nZEB) is difficult. These authors analyzed nZEB definition from various perspectives and proposed a methodology for setting ambitious but realistic primary energy requirements considering a large sample of buildings (Szalaya and Zöldb 2014). Presented method is an example of setting the requirements for residential buildings in Hungary. Proposed requirements were validated against the common European targets. The conclusions state that it is necessary to formulate the requirements which are technically achievable. There also exists a possible different approach using simulation-based multi-criteria optimization of NZEBs (Harkoussa et al. 2018). This methodology is a useful tool to enhance nZEB design and to facilitate decision-making in early phases of building design.

A separate issue is the modernization of buildings to nZEB standard according to already established definitions and criteria (Brambilla et al. 2018). The renovation and reuse of the Atika building, a demonstrative energy-efficient building, is an interesting case. It is presented as case study of an environmental-efficient methodology for energy retrofitting. In order to achieve set goals, the researchers found the necessity for integrated design methodology. The example shows that it is possible to optimize the choice of materials and installed equipment based on the relationship between the energy spent in the construction and the energy saved during the operation. In a modernized building, there is always a possibility to reuse the existing structural elements, as this choice has a positive influence on the LCA, as well as circular economy of building materials. This issue was discussed during research and evaluation of thermal comfort in domestic zero-energy buildings (Pomfret and Hashemi 2017). Dynamic simulations were used to assess various scenarios possible in a low-energy dwelling including type of building’s structure, natural ventilation strategies, solar shading, and occupancy periods. Thermal comfort also appears to be an important issue that should be included not only in new, but also modernized buildings.

In nZEB buildings, it is extremely important to use non-standard solutions such as individual ventilation systems, which may influence internal air temperature reduction in winter and increase during the summer, and in turn, affect the total building energy consumption (Chludzińska and Bogdan 2017). In case of the Powerhouse Kjobro building modernization, the radiators were located in the central part of the building on the corridors, so in order to heat the rooms located next to the external walls, the offices’ door had to be open in order to allow for infiltration of heated air. This in turn influenced user behavior (Sørensen et al. 2017). Hence, it may be stated that the efficiency on nZEB buildings also relies on the user knowledge.

## Methodology

Already mentioned, recast of EPBD (EPBD Recast, Directive 2010/31/EU) also had impact on the research initiated 2015–2017 by two European Universities and interdisciplinary tasks undertaken by the researchers. Hence, “Design retro-fit nZEB concept for two buildings – KODnZEB”—a project which was developed by Warsaw University of Technology and NTNU Trondheim, based on EU financing—actually falls in line with the contemporary scope of developments in various countries (Karima and Altan 2016), but the approach has to differ due to existing legal procedures and technical conditions.

According to EPBD Recast (EPBD 2010): “nearly zero-energy building means a building that has a very high energy performance” and “energy required should be covered to a very significant extent by energy from renewable sources”. The nZEB standard is achieved when Primary Energy Factor (PEF) value is higher than in a zero-energy building and lower than in a building, which meets national minimum requirements. In KODnZEB project, nZEB building was defined as the one, which meets 90% of requirements for a zero-energy building (Kwiatkowski et al. 2017). This definition was chosen, as presented within this paper, analyzed building was undergoing retrofit development and it was decided to lower the existing new building benchmark. Thus, PEF values for nZEB are as follows:

- for collective dwelling building (without cooling) 9.5 + 5.0 kWh/m^2^/year
- for collective dwelling building (with cooling) 9.5 + 5.0 + 2.5 × A_f,c_/A_f_ kWh/m^2^/year
- for public use building (without cooling) 6.5 + 10.0 kWh/m^2^/year
- for public use building (with cooling) 6.5 + 10.0 + 2.5 × A_f,c_/A_f_ kWh/m^2^/year

where A_f_ is heated usable area and A_f,c_ cooled usable area.

In addition, the values of heat transfer coefficients cannot be higher than 0.2 W/m^2^/K for external walls, 0.15 W/m^2^/K for roofs, 0.9 W/m^2^/K for windows, 0.3 W/m^2^/K for ground level slab, and 0.25 W/m^2^/K for ceilings slabs over unheated spaces. Energy balance was calculated yearly for the whole building (building area is calculated to the outer façade with installations) and includes primary energy from heating, cooling, ventilation, warm water heating, lightening, and auxiliary energy. Calculations were made in accordance with PN-EN 13790 (Kwiatkowski et al. 2017) and with the use of Design Builder Software. Research included possible energy saving which could be achieved through the use of green walls inside and outside the building. This solution was analyzed in the Jubilee Campus of University of Nottingham, where the thermal regulation feature of green wall systems was experimentally and numerically investigated (Cuce 2017). The outcomes were that an external green wall with a thickness of 10 cm provides an average reduction of the internal wall temperature by 2.5 °C. Many other researchers have also described the influence of green areas and green walls on the well-being, internal comfort, and energy demand (Ling and Chang 2018; Perini et al. 2017). Internal green walls are not only a design element, but also provide benefits on human’s health and productivity. Some of them remove carbon dioxide, minimize dust, reduce level of pollutants, and assist in minimizing the effects of a sick building syndrome (Raji et al. 2015). Plants can also increase the average humidity of the indoor air which may enhance the thermal comfort of the occupants (Lohr 1992). Positive influence of an indoor living wall on the temperature and humidity was also confirmed in research carried out in the hall inside the School of Agricultural Engineering (University of Seville) (Fernández-Cañero et al. 2012). Energy savings can be achieved due observed cooling effect of the living wall, which in this case proved reduction of average temperature by 4 °C in the green wall’s vicinity.

It should be noted that for the purpose of the grant, the design team (circa 30 designers of various disciplines) decided that the accomplished solution, based on design calculation, would allow the building to achieve U = 20 kWh/m^2^ year, whereas according to official technical Polish requirements, nZEB public buildings are considered from U = 120 kWh/m^2^ year threshold. We also assumed that in future, we will be able to construct one of the buildings as a demonstration building where more scientific research could be conducted and rechecked.

Hence, since national threshold appears to be high in comparison with other countries, it was decided that the Kjorbo building will be regarded as a reference building for the purposes of this grant.

**Fig. 1:**
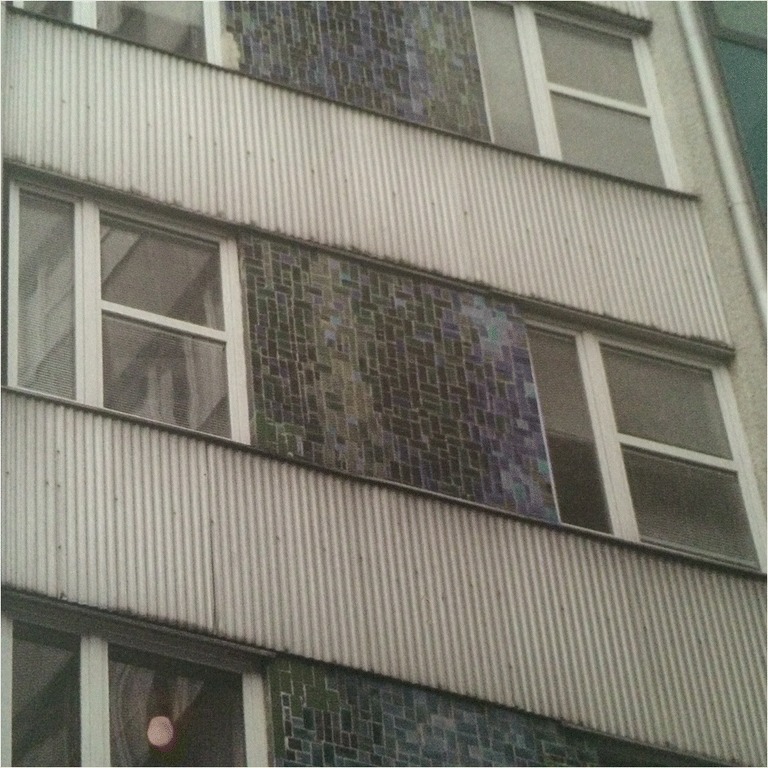
Facades of the Faculty of Building Services, Hydro and Environmental Engineering (KODnZEB, 2015–2017)

Research outcomes were provided in several work packages dedicated to different research areas. In case of this paper, only some issues will be presented. The first one revolves around initial management procedures required to provide an nZEB interdisciplinary design aiming to provide retrofit solutions of existing public building, with emphasis on the possible use of management procedures and technologies used in Norway for that purpose. The second work package presents a case study prepared as one of the grant’s achievements. Applied design procedure is based on the management scheme envisaged in the research. The key issues are included in the energy analysis of one of the case buildings, its outcomes and impact on the final architectural choice. The retrofit design of the Faculty of Building Services, Hydro and Environmental Engineering (FBSHEE) Warsaw University of Technology building to nZEB level has been prepared according to the Polish Building Permit standard. The chosen site is located in the Main Campus area listed as a historic preservation zone, but the building itself is not under historic monument protection. It was constructed in the 1970s of the twentieth century, based on design by S. Jaczewski and J. Reda. It is “L” shaped, forming an internal open atrium set against existing early twentieth century historic building. The wing located parallel to the main street is eight stories high; the perpendicular one has eleven levels. It is used on everyday basis by different groups of students and employees (circa 2000 people). Main load bearing elements are of reinforced concrete, monolithic in the basement level, and prefabricated slabs and framework skeleton elements on the upper floors. Architecture features (glass inserts in various shades) form a strong contrast with surrounding historic buildings (Wagner 2015). FBSHEE building is a case of a standardized prefabricated concrete structure—and unfortunately—also an example of low technical and construction standards, later on followed by a permanent state of inadequate maintenance and financing (Fig. 1).

## Preconditions for nZEB upgrade development

Preconditions for the nZEB development were set forth within the KODnZEB grant, in the Workpackage headed Management. It was assumed that in environmental construction process, and especially when designing buildings classified as nZEB, the most important phase is the Concept level and initial Brief Conditions, when most of the key decisions for the whole life cycle of the building including function and maintenance are considered. This especially includes procedures to be used both by the client (in this case, representatives of the Warsaw University of Technology) and representatives of all design disciplines when working towards achievement of proposed aims. A preliminary design specification defining the scope of the services to be provided “for” and “in the name” of the client was considered as a very helpful document, prepared in cooperation with consultants from different disciplines. Scope of tasks was described in accordance with the designer’s best knowledge and client’s expectations, accepted standard of the investment and bench marked internal environmental parameters, time schedule, and existing budget. All data and additional requirements initiated during workshops with the client, including environmental, urban planning, and financial effects were included as part of the project’s evolution. Explanations concerning the level of durability characteristic to the chosen building materials, as well as efficiency of designed installation systems, energy strategies were discussed as part of the design process. This procedure also has been confirmed by other researchers in various countries (Fouche and Crawford 2016; Karlessi et al. 2017).

It should be noted that one of the most neuralgic zones within the building investment is design process which in many countries has been treated as a linear system, where the lead designer—usually an architect—presents a stand-alone aesthetic solution to be consulted by other disciplines. Unfortunately, representatives of those “other” disciplines also tend to work separately. Therefore, this chosen strategic management included a modification within design process procedures. The main idea is that all participants should understand that technical solutions must be perceived holistically—that efficient energy use is not a priority when there is insufficient user ventilation level at stake, or low standard of internal use environment. Furthermore, buildings should be perceived as one of many elements of the urban context and their influence on natural surroundings and already existing urban space must be analyzed (Liu et al. 2016). Impact of existing climatic parameters on the form, functional layout, and choice of building materials and technologic systems is also a priority.

It may be therefore assumed that any issues concerned with the buildings where sustainable development strategy was used, concentrate not just on the ecosphere, but also on people and economic perspectives. Hence, the perceived scope of potential interests is *Efficiency* of used tools and solutions, in balance with *Ecosphere*—scoping both humans as well as well as any biotic and abiotic species. Both areas should be joined together with *Economic* abilities, not always articulated as financial gain, but accepted as a complex phenomenon mirroring human requirements. In fact, a *Triple E* was assumed throughout the design (Rynska 2018).

Research proves that additional cost for design and construction phase should not exceed 12.5% of total costs even in case of nearly zero-energy buildings (nZEB). In case of those buildings, efficiency of costs was influenced, but not be limited to (Rynska 2018):

- Acceptation and implementation of environmental solutions at early design stages; design strategy must include preliminary cost estimation.
- It is preferable that design team will use integrated design process, allowing connection of energy-efficient passive envelope solutions, layout of function with regard to required energy needs, and possible choice of high-efficiency equipment which may be used.
- Employment of design teams which have experience and use integrated design process, as well as implement PBBD or BIM management already at early concept stage, through all stages including user participation.

Utilization of integrated design appears to be an issue not only because of environmental assets, but also due to economic feasibility of designed buildings which will bring long-term financial gains and economically satisfactory costs, due to implementation of durable building materials and equipment.

Part of research included review of Directive 2010/31/EU (Kwiatkowski 2017) (Directive 2010/31/EU), which conforms that it is the responsibility of the Member States to set minimum energy performance requirements for building and building elements and that the requirements must be set with a view to achieve cost-optimal levels. Proposed calculations of global costs for financial calculations contained in Annex 1 to this Directive was too complex to be used at a concept design level and it was indicated that the initial calculations should be provided based on net present value (NPV) method determined by calculating the costs (negative cash flows) and benefits (positive cash flows) for each period of an investment. After the cash flow for each period is calculated, the present value (PV) of each one can be achieved by discounting its future value at an annual rate of return. NPV is the sum of all the discounted future cash flows.

So what were the strategy preconditions set for retrofitting developments to nZEB standard?

One of the main issues was the analysis of economic feasibility of chosen solutions. Since cost calculations do not form part of this paper, only general conclusions and procedures will be presented. First, assumption was that cost difference of design and construction of a new building in standard and nZEB level should not exceed 12.5–15%. Since FBSHEE is an existing building and will undergo modernization to the described standard, after interdisciplinary discussions and review of the building’s existing solutions and technical state which included description of additional construction works which had to be provided in order to allow nZEB solutions, the final cost difference was established at 13.5–17%. In order to maintain assumed economic level, cost estimations were repeated with each interdisciplinary modification introduced into design. Final cost estimates were used in order to calculate return of costs which included discount factors (initial discount factor for May 2015 was set as 3.16 according to official information). Calculations showed that the return of costs payback period should not exceed 7–8 years, based on the assumed lower level of facility management cost involved after modernization.

When preparing analysis of the local characteristics, designers maintained a holistic management strategy. One of the major conditions was that analysis of urban space was considered as an area larger than just the site, and each of existing ecosystems was checked in relation to all neighborhood ecosystems. Hence, “environment” was understood within a global context. All proposed nZEB solutions included not just economic- and energy-efficient parameters, but all feasibility abilities of actual site and health and well-being of the users.

Complex management of environmental construction investment process, with a particular consideration of nZEB solutions, did not mean preservation of the environment from human intervention, but rather creation of relations between the effects of the human intervention within the manmade and natural ecosystem and the environment itself. It concentrated on finding ways of reducing negative influences due to ineffective energy solutions (Rynska 2008). Checking procedure started already during the predesign concept level. The set of issues within the design and construction phase scopes most evident contemporary choice—efficient energy solutions—as well as efficient use of building materials, water, and soil.

In the case of the FBSHEE building, simultaneously with the initial economic analysis, researchers prepared and distributed amongst building’s users (both students and academic teachers) a questionnaire containing 38 questions which released information on the user awareness of various issues. The outcome was that the main positive feature of the site is good access to public transport (trams and busses), as well as proximity to other amenities surrounding the Main Campus area. Aesthetic qualities of the building’s form and internal solutions were perceived negatively (circa 65% of respondents). More than 90% of respondents pointed out that major modernization of the building was required, especially for lecture rooms as well as common areas and sanitary units. The layout and orientation within the building was estimated as average. Many respondents commented on the technical state of lifts, inadequate access for persons with limited mobility, and illogical functional division of the 6th floor where some of the areas can be accessed only through separate staircase. Nearly 80% of the respondents confirmed adequate size of the teaching areas; this was in contrast with 60% of respondents who remarked on nonexistence of spaces for individual studies and recreation. Internal comfort parameters were estimated as average—this appeared to be similar for the access to daylighting and electric light system, air quality, and the level of cleanness. Circa 60% voted for sustainable solutions, with over 90% supporting the choice of photovoltaic panels on the facades with South exposure. Last renovation works (2014) included roof insulation of the lower building, replacement of old windows, installation of a small area of photovoltaic panels (circa 60 m^2^) on the Southern façade, and minor fit-out changes in general access areas. Unfortunately, new solutions do not improve architectural features. Energy audit showed that additional new insulation layer is required for the building’s roof and elevations. Necessary modernization should not be limited to insulation only.

Following priorities were established:

- Modernization of fit-out, sanitary areas, and furnishing
- Modernization of elevations
- Provision of adequate heating and cooling comfort standard
- Provision of recreation and individual study areas
- Exchange of ventilation system and light fixtures in order to achieve expected user standard
- Provision of handicapped access and use of all building’s areas

Further assessment was made during various visits into the building and surrounding site, as well as interviews with the building’s facility management. Hence, the user requirements became one of the aims within the research and proposed final solutions.

The initial economic choice was to continue education process during modernization, as this solution was 30–35% cheaper in comparison with renting costs if teaching was to be periodically moved to a different building, as well as involved reuse of existing structure and some of building systems. This meant that designers had to form a multipurpose “second skin” mounted on existing facade. This skin integrated energy efficient systems as well as aesthetic measurers.

When optimizing design process, the designers were obliged to formulate a set of criteria which included, but were not limited to (Rynska and Kozminska 2018):

- Highly insulated building’s envelope and energy efficiency of chosen installation systems.
- Low environmental influence of chosen building technologies as well as choice of fit-out materials with lower environmental impacts.
- High standard user environment.
- The best case scenario prepared with data including durability and efficiency of chosen building materials and installation systems.
- Easy access to equipment and effective performance of installation systems.
- Cost investment efficiency, additionally supported by LCA analysis—both prepared and updated during each stage of design process.

Within integrated design, traditional phases remained unchanged. The only difference was positioning of the multidisciplinary process in an interactive “loop”, where each of the architectonic and technical issues was optimized during repeatable consecutive phases of the design process. The key aspect was constant cooperation between representatives of each of the disciplines, interdisciplinary integration of proposed solutions, as well as workshops with the building owner. Design documents prepared according to Integrated Design Conditions (IDC), allowed achieving additional efficient energy use, while maintaining the same level of construction costs. It has also been practically checked from experience and other projects (Rynska and Bartkiewicz 2017) that the best phase to include nZEB solutions is the concept phase. This condition complements BREEAM requirements. Hence, it should be noted that when using IDC, following issues were included:

- Interdisciplinary cooperation starting from design concept level, as only this type of approach allowing for a holistic determination of benchmark parameters and realistic estimation of costs.
- Appointment of a dedicated project manager—a person, who participated in all meetings and played a management key role within the process of attaining a coordinated set of interdisciplinary drawings and specifications.
- Introduction of final technical parameters defined for each of the disciplines, and holistic and economic parameters which will have to be fulfilled by the building and checked when operational.
- Cooperation with environmental, energy, and quantity surveyors preparing initial cost estimation—starting at concept design stage
- Preparation of main environmental strategy conditions, allowing for a framework where other specialist consultants such as daylight consultants, may work on the optimization of the daylighting, others may be consulted on air quality; each of those consultants may be approached to formulate an opinion during all design phases.

Many of the above conditions appear to follow a typical design process. The main difference may be perceived in the fact that both the client and/or the end user play the key roles during design process. This is especially vital in nZEB buildings which evolve around energy strategies. Hence, when establishing the initial brief, designers considered following issues (Rynska 2018; Sowa 2017):

- Type of build-in materials and surface finish to be used on the external face of the building’s envelope; it was essential to check daylighting options and passive heating solutions of the building’s internal areas, use of interactive outer-skin facades should consider cooperation between environmental parameters which are compensated by man-made technical systems.
- Choice of heating, ventilation, and air condition systems—use of efficient energy solutions, natural ventilation systems, and securing alternative energy sources.
- The choice of the daylighting strategy—level of artificial light system should compensate the existing fluctuating level of daylight—in total fulfilling demanded level of lux. Artificial lights should be switched on only in the areas in use (unless the user technology demands otherwise).

Building and finishing materials specified by the designers had the “eco-friendly” status established through following criteria (Rynska 2018; Rynska and Kozminska 2018).

- Low energy use and low primary energy during manufacture and in-build processing of building materials.
- Manufacture process of building materials and components from renewable or recycled resources or components, also allowing use of recycled materials from consumption and industrial waste.
- Maintaining good standard natural environment parameters—low emission of greenhouse gases.
- Use of materials and technologies characterized by low or no emission of dangerous substances which might be emitted during production and in-build process, as well as at the end of technical life.
- Choice of building joints allowing for recycling of elements or dismantling into basic components which can be used in a different location or reused in a new building process.
- Technologies using easy mounting techniques, without need to use additional volatile components.
- Building materials produced in locations at rationally near distances from the site; this also has impact on the lower use of vehicular transport, level of emitted energy, and emission of greenhouse gases.
- Building materials and equipment should be packaged in materials which can be resourced and reused or recycled.

When dealing with the choice of materials in Poland, the researchers found that choice of green materials was an issue, as most of the approached national firms had very little knowledge on those matters. Hence, some of the materials indicated in Technical Specification had to be of foreign origin.

Additionally, when using Integrated Design Strategy, designers presented the client with data containing program analysis, estimated costs, and initial bill of quantities. Due to legal conditions, basic environmental solutions included (Communication from the CEP 2008) environmental-friendly building materials, energy calculations for the building’s volume, and calculations containing thermal capacity of building elements. When analyzing most efficient structural solutions, lowest primary energy and life cycle was assessed.

## nZEB retrofit case study

In existing conditions, the primary energy requirement in analyzed building highly exceeds the coefficient established within the KODnZEB aims as 20 kWh/(m^2^ a). The initial conditions set forth included the scope of modernization works which not only would reduce the heating, cooling, warm water, and electric light system energy requirements, but would also include a higher standard of the user heating comfort expectations. Heating comfort was analyzed according to the EU harmonized standard (EPBD 2010), (PN-EN 15251).

Contemporary layout of functions places education, laboratory, and office areas located off South and West facades in a zone highly overheated during the summer months. Additional problem arises from the high level of traffic noise—a tramway and a double lane road—located 30 m from the building’s front line.

Hourly analysis of the building’s energy needs was prepared with a program for holistic heating building analyses DesignBuilder version 4.2.0.054 (DesignBuilder version 4.2.0.054.), using nationally accessible climatic data (PN-82/B-02403). This program fulfills the CIBSE AM11 “Building energy and environmental modelling” conditions. Geometric information and building’s parameters, including heat transfer coefficient U (external walls 0.20 W/m^2^ K, roof 0.15 W/m2 K, windows 0.9 W/m2 K) solar radiation transfer coefficients g, were part of the input to the chosen program. In the next step, model was updated with data concerning characteristic building zones—number of potential users, strength and quality of accessible light, level of ventilation input, location of equipment including their profile use. Building’s final model contained 237 zones, over 100 schedule definitions and circa 200 HVAC systems. This detail of the used model allowed calculating simulated energy use in different zones for the heating, cooling, light, warm water, or additional drive’s requirements.

**Fig. 2:**
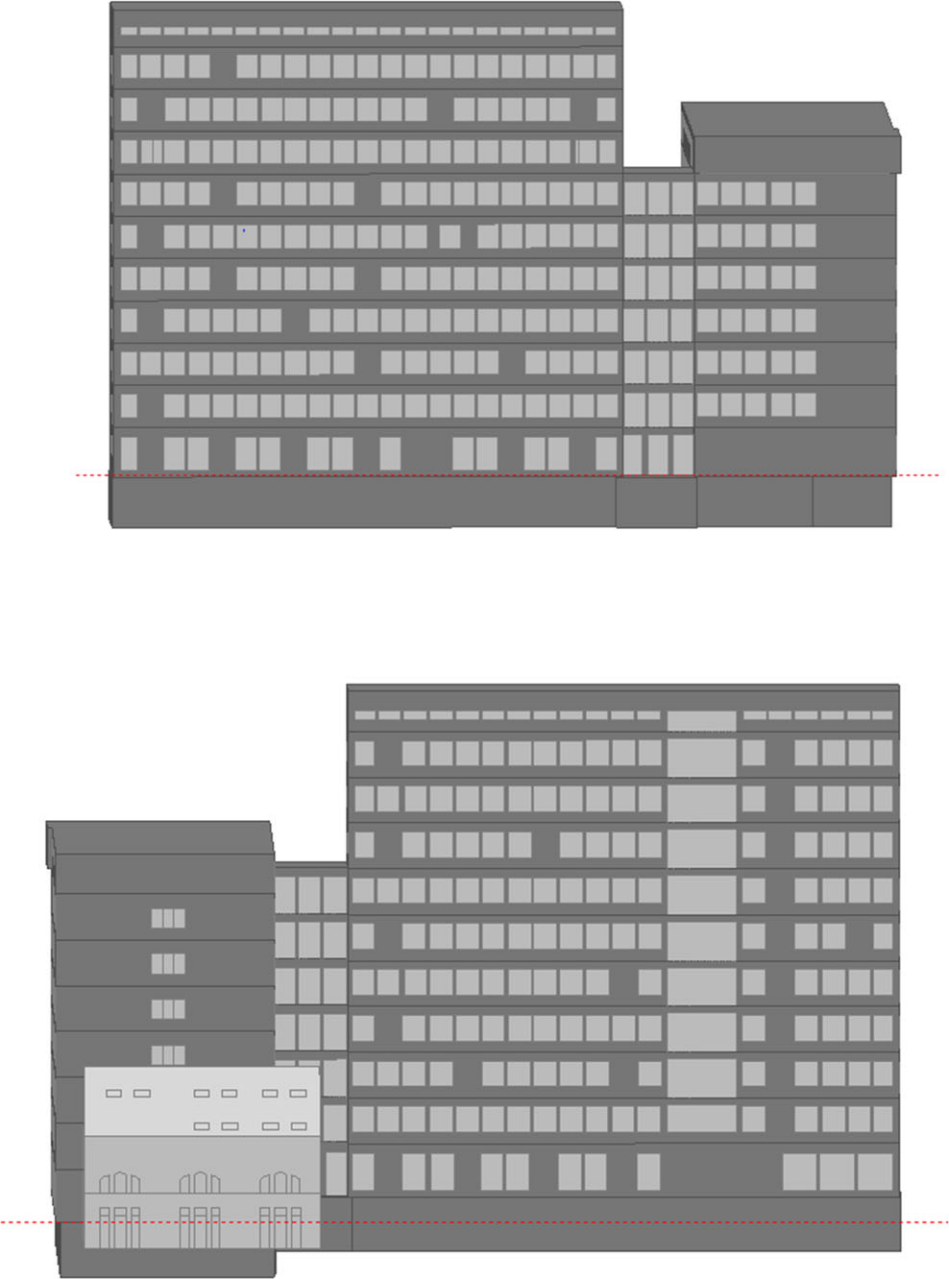
View of facades (west facade above, east below)—model of existing building FBSHEE

**Fig. 3:**
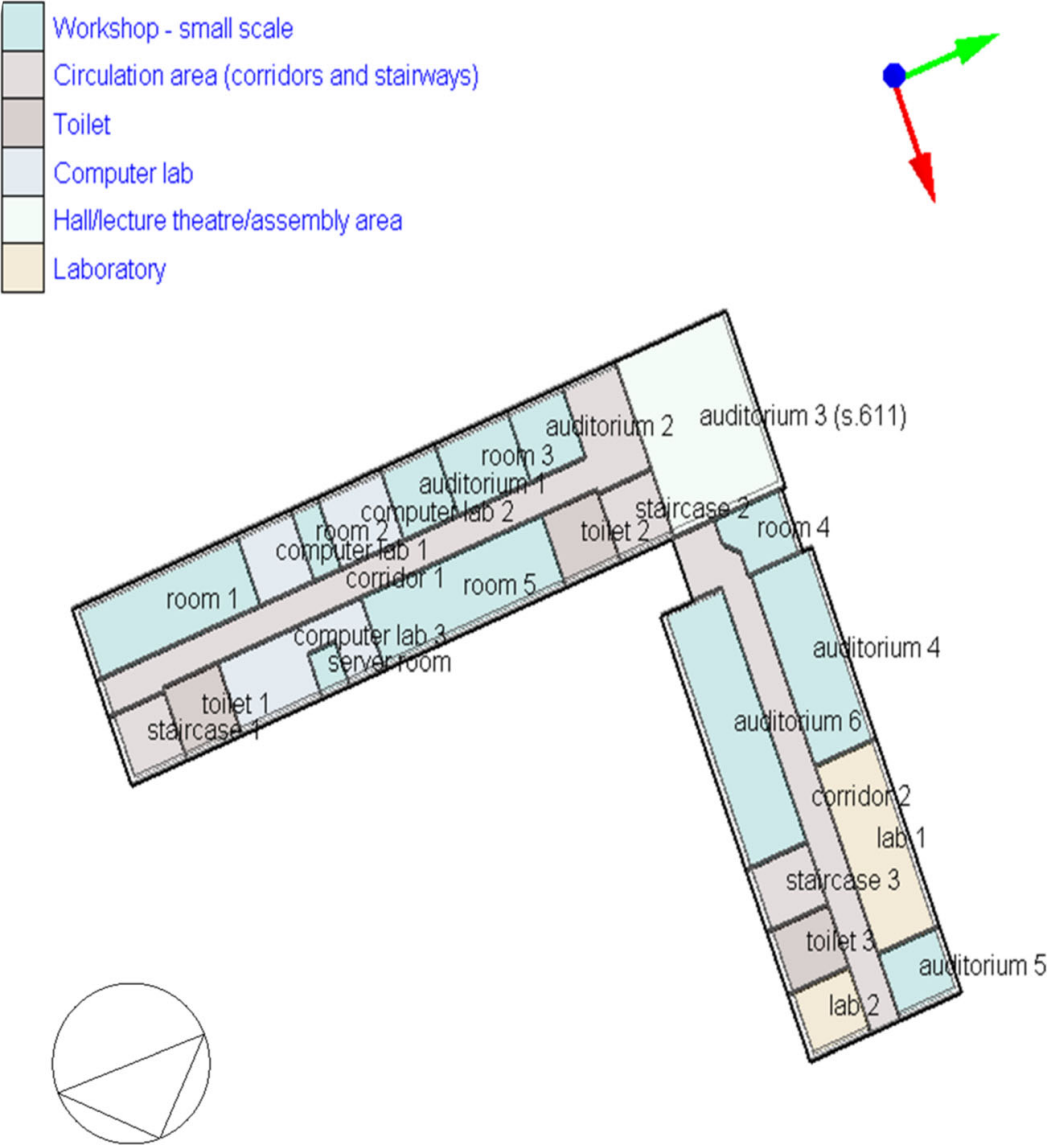
Level 5 (+4) model of FBSHEE building

**Fig. 4:**
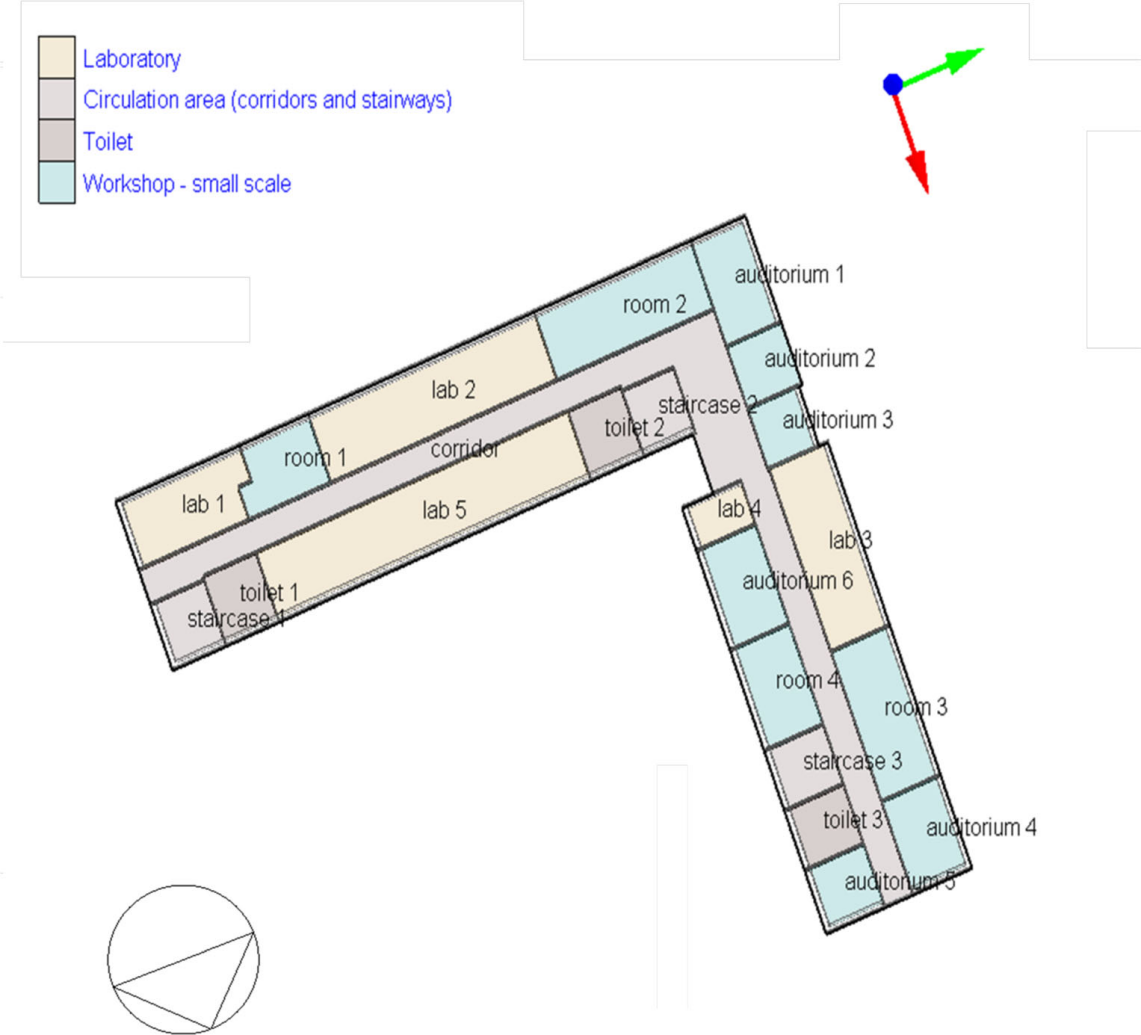
Level 7 (+6) model of FBSHEE building

The initial phase included analysis of the building’s existing state. This model was verified through comparison between calculation output and real measured data (Fig. 2). Plans of the building levels with zoning are shown on Figs. 3 and 4.

**Fig. 5:**
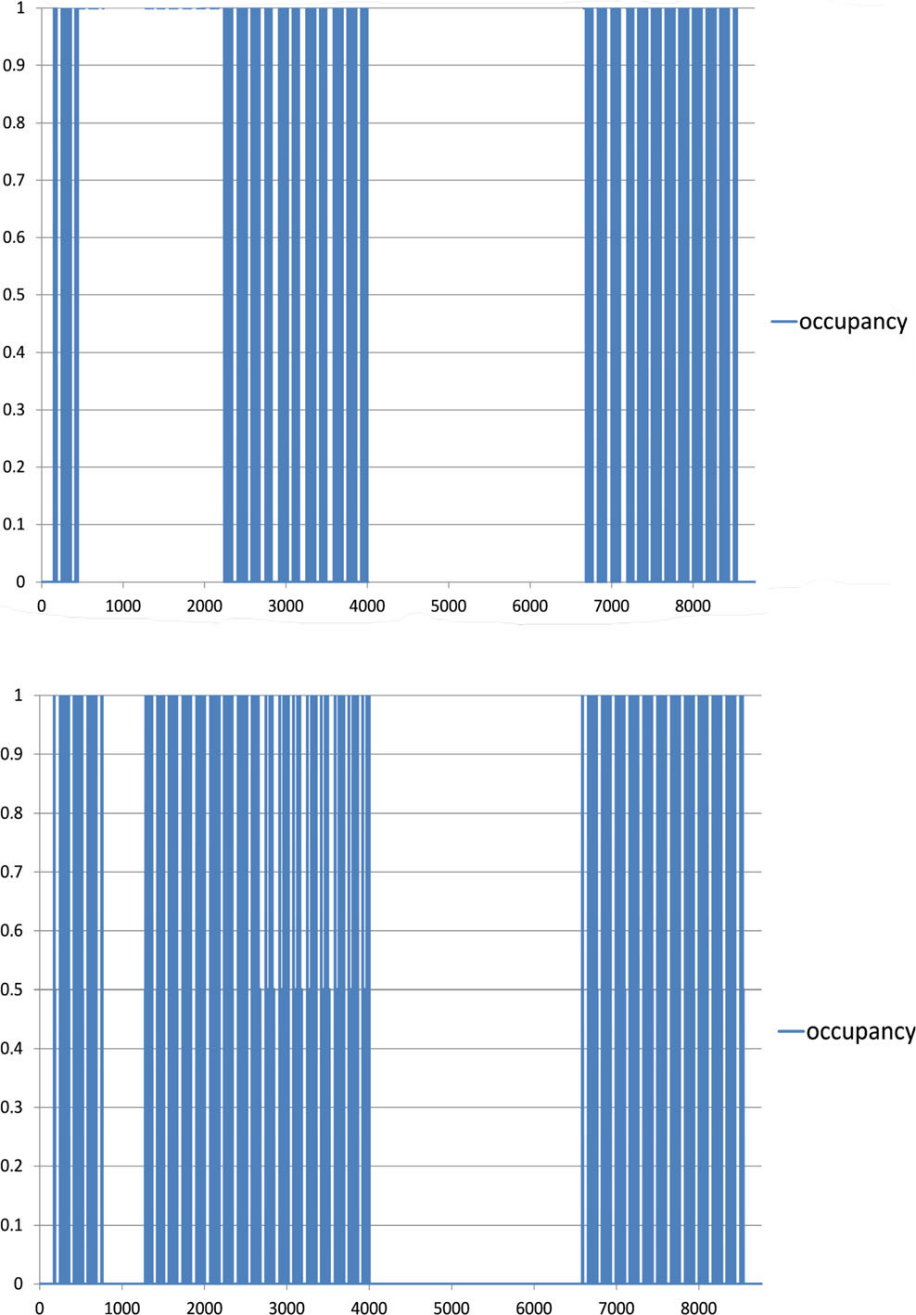
Use schedule (auditory hall no 415 above and auditory halls 627 + 629 below)

An example of chosen teaching areas’ annual usage is shown in Fig. 5. Schedules are described based on the actual use of individual areas according to the FBSHEE official time table.

**Fig. 6:**
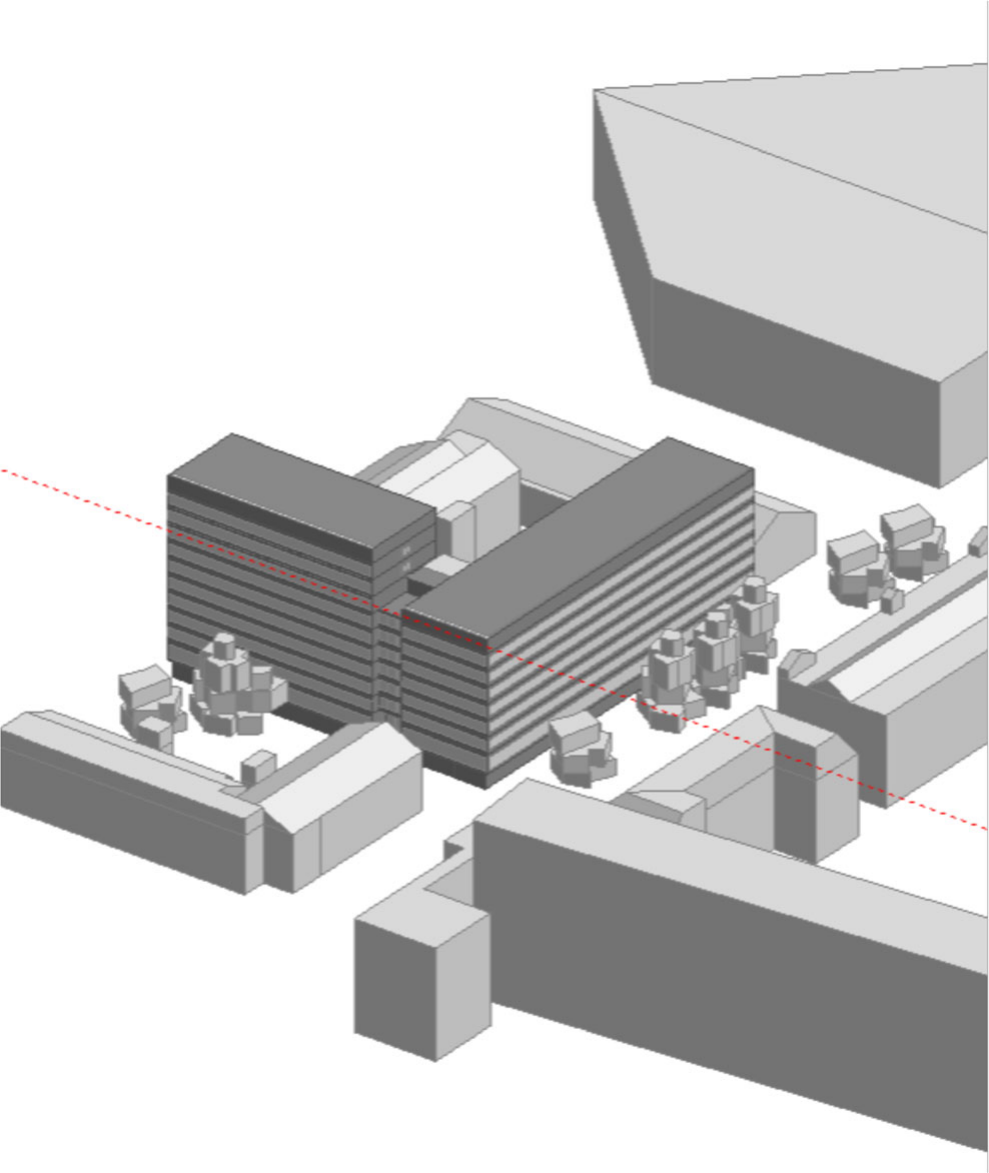
Model view of the FBSHEE building, off south-west side

After verification of the model’s results within the integrated design process, researchers chose modernization solution allowing limitation of the energy needs through lower heat losses through building’s external envelope and introduction of air ventilation preparation process (Integrated Design (2012–2014). Based on the energy analyses and individual rooms’ thermal comfort analysis, the case best alternative was chosen. This meant better insulation of the external building elements, fitting out the ventilation system with input-output and heat recuperation units, including adaptation of the air flow streams to the number of users, as well as modernization of the light fixtures in order to reduce energy demand. Model of the FBSHEE building is presented in Fig. 6.

**Fig. 7:**
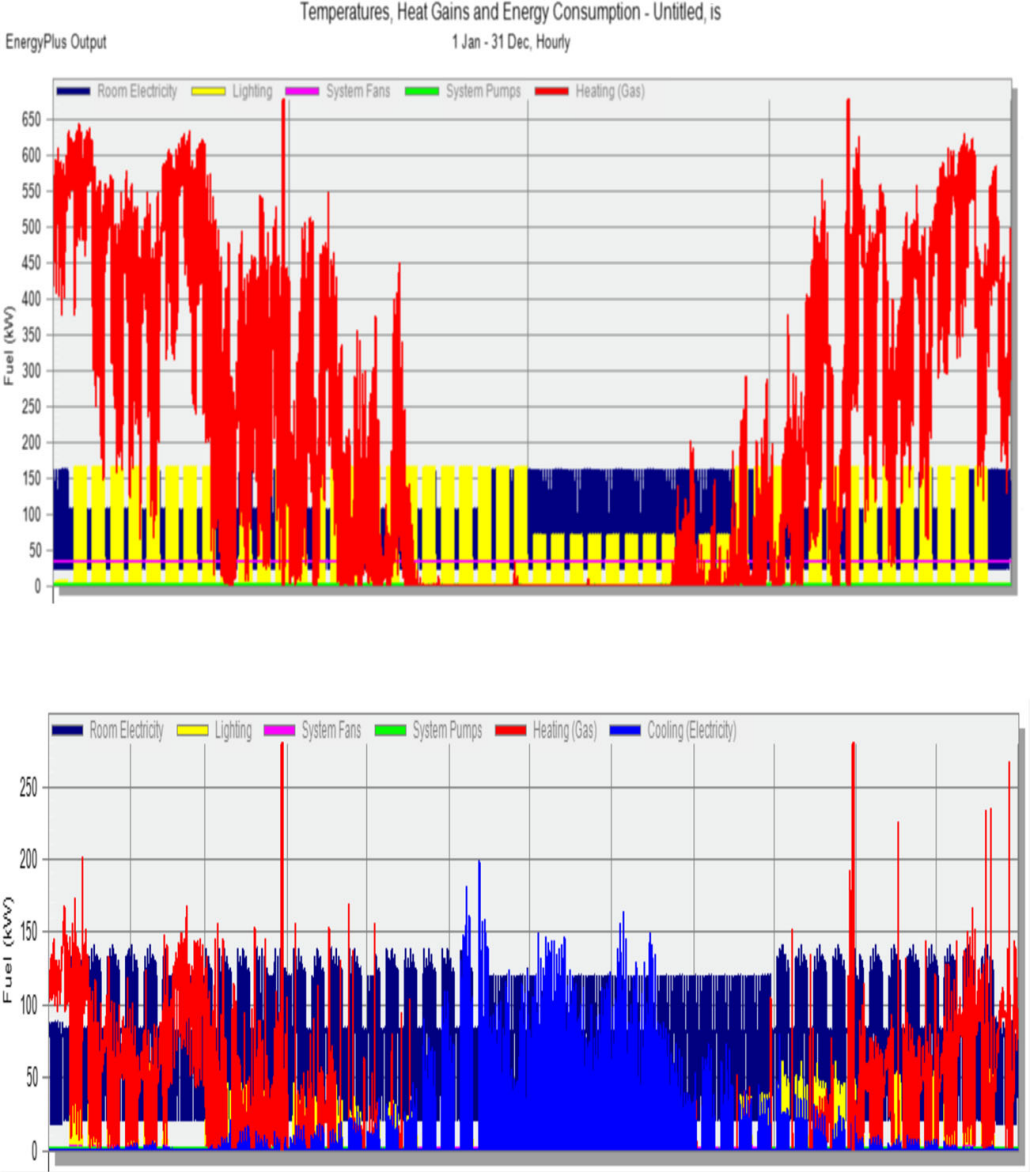
Presents hourly graph of the of analyzed building’s energy needs in existing state and after foreseen modernization, which should allow lower power requirements. Hourly energy requirements in FBSHEE building (above—prior to modernization, below—after modernization)

Analysis conducted during design phase established source of the heating supply. Presently used heating source (city grid) has a low coefficient of alternative primary energy sources; hence, the choice of potential sources allowing for a better primary energy coefficient was very limited. It was decided to use following sources: city grid (covers 21% of heating needs), ground heat pumps (as support medium, covering 6% of the heating needs), and a micro-generation unit (co-generated heating and electric energy output covers 73.9% of the heating requirements) (Fig. 7).

Table 1 contains the values for alternative primary energy required for the building prior to and after the foreseen modernization process proposed within the KODnZEB design.

**Table 1:** Primary energy requirement coefficient kWh/(m^2^year) for the chosen alternative solutions

System	Primary energy requirement coefficient kWh/(m^2^rok)	
	Existing	After modernization
Heating and ventilation system	72.2	3.50
Support units in HVAC system	12.1	11.7
Usable warm water	8.8	2.6
Support units in usable warm water system	0.7	0.5
Cooling system	0.9	4.9
Light system	60.6	10.5
PCV panels	− 6.5	− 27.5
Total	148.9	6.2

Building energy characteristic achieved after proposed modernization process based on the analyzed energy simulations fulfilled the requirements posed for the nZEB buildings within the initial KODnZEB conditions.

Conditions accepted in this project are stricter in comparison to the country’s national legislation requirements for building heating preservation needs (National Plan 2015; Legal Act 2012) and based on Kjorbo building solutions.

**Fig. 8:**
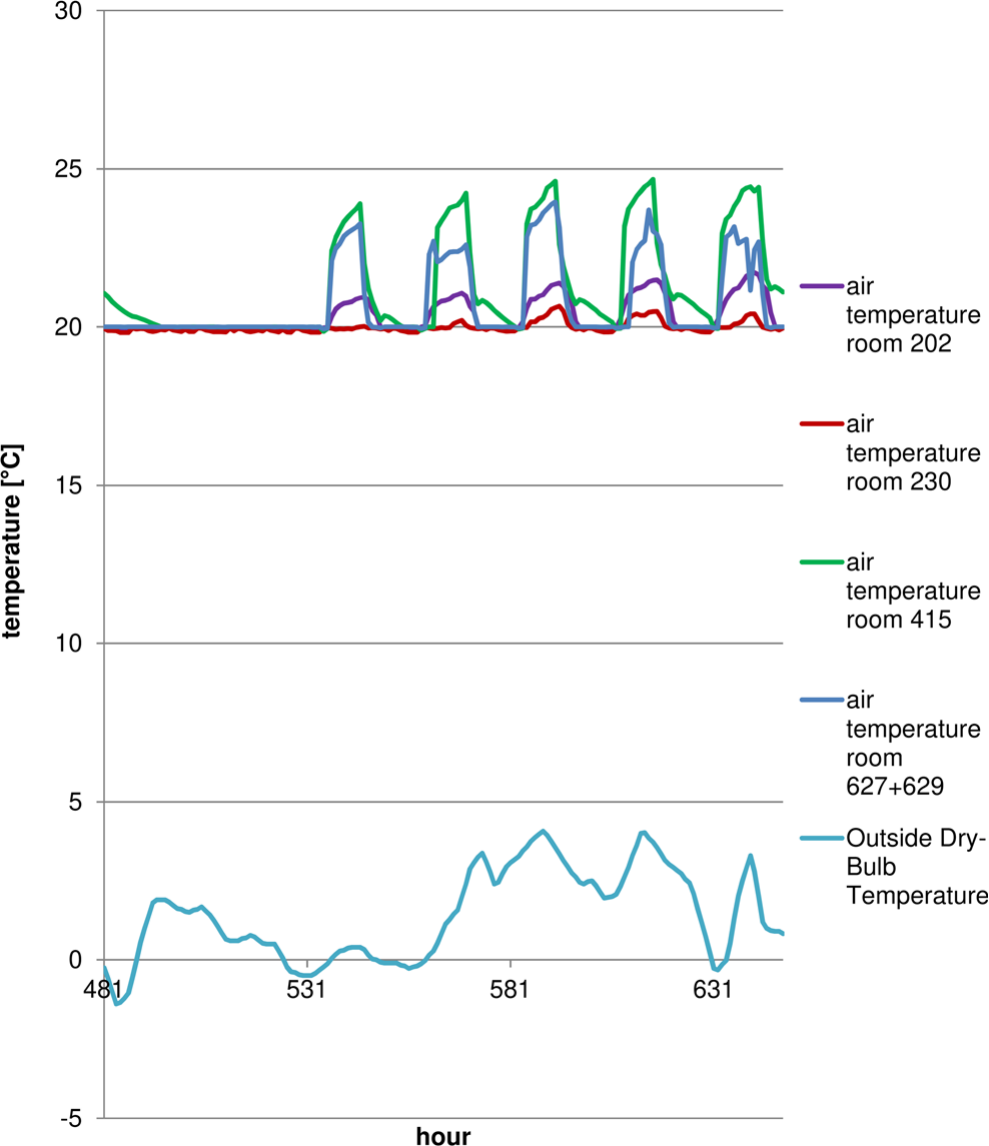
Temperatures in a chosen week (winter)

**Fig. 9:**
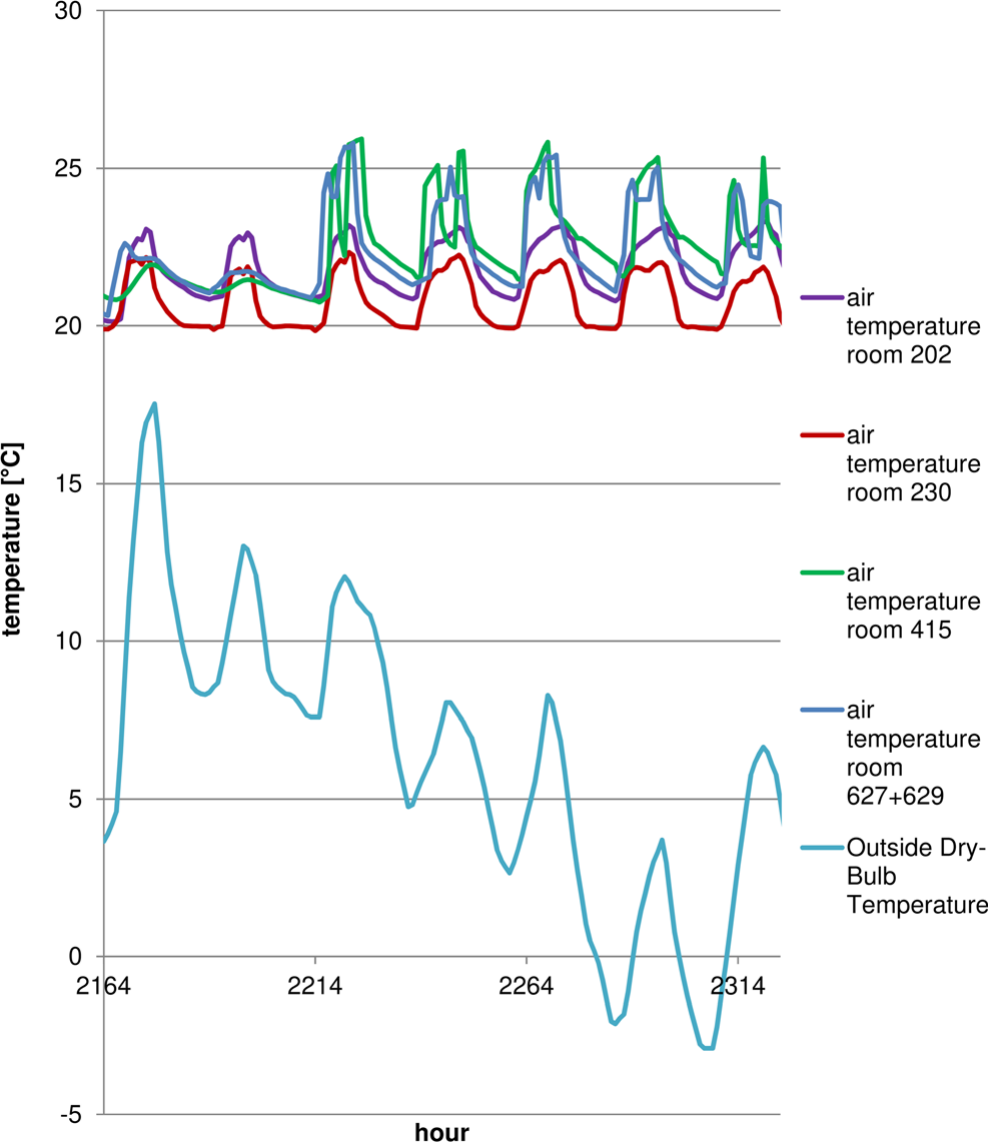
Temperatures in a chosen week (spring)

The model shows that during the winter, auditoria and teacher offices will maintain set temperatures. Overheating does not appear during the summer months in most areas (Mijakowski et al. 2017). It should also be mentioned that the building is hardly used during the warmest months (summer holidays lasting July through August). Figures 8 and 9 present temperature distribution in chosen areas (room 202 and 230—office rooms, auditoria no 415 and 627 + 629). It should be mentioned that in order to comply with CIBSE TM52, rooms 202 and 230 (where summer internal temperatures approach 32 °C) were additionally fitted with a cooling system, maintaining the temperature below 24 °C. Rooms 415 and 627 + 629 are auditoria, not used during summer months; therefore, if required, reduction of temperature in these rooms is possible by periodic higher values of the ventilation rates.

Additional added value arising from the modernization is lower emission of CO_2_ to atmosphere. Total emissions of CO_2_ from analyzed systems in existing and post modernization state are shown in Table 2. Calculations were prepared with emission coefficients chosen from the webpages of the National Centre for the Emissions Equalization and Management (KOBIZE).

**Table 2:** CO_2_ emissions

System	Emission coefficient CO_2_ [tCO_2_/MWh]	Emission CO_2_ [tCO_2_/a]	
		Existing state	After modernization
Heating grid	0.229	497.67	20.51
Electric energy (heating pump)	0.812	–	1.32
Electric energy (electric energy network)	0.812	262.34	434.96
Gas	0.202	–	61.52
Co-generated production	0.812	–	− 123.64
PCV production	0.812	− 34.04	− 165.19
Total	–	852.70	229.47

Total CO_2_ emission coefficient prior to foreseen modernization was calculated at 44.1 kg CO_2_/(m^2^a); after foreseen modernization, it should be lowered to 11.9 kg CO_2_/(m^2^a).

During the grant’s development, the main aims were also health and well-being solutions, as well as provision of aesthetic choices simultaneously merged with strict technical conditions. Therefore, from the start, it was obvious that facades were to be redesigned: a curtain wall with photovoltaic panels was added to the south and west facades. This was done after careful planning as to the type of materials used, level of efficient energy requirements, and as well as user comfort concerning the protection from overheating and the view out. The glass shell with independent steel structure is placed at a 1-m distance from the existing reinforced concrete wall. The existing wall is insulated (21 cm of mineral wool, double-glazing of existing walls with mosaic detail), plastered, and covered with ventilation ducts. The curtain wall hides installations, protects from the external noise, and integrates elements, which shade and produce energy. Photovoltaic cells create the detail of the façade. They are more densely spaced in horizontal area between windows, where they cover ventilation ducts. There are fewer cells in the window area, upper part of the façade, and on the first floor level to create blurred edges of the effective (in terms of energy production) elevation zone. Similar solution—blurred edge of cells create a transition between high-tech southern façade and the historic Old Boiler House—to underline the contrast between the old and new substance (one of the requirements presented by the historic monument protection analysis). Aluminum sheet cladding with in-built pots for greenery covers the north and east part of the building. This choice was a result of the analysis of economic possibilities, technical requirements, and achievement of an internal green courtyard which would become a heart of the building. Horizontal ventilation ducts, placed between windows, are flattened in depth, reduced to 20 cm with an adequate cross-section area still maintained (required relation between width and height of the duct is 1:5). This modification of ducts enabled to hide them and the layer of insulation under the customized aluminum cladding. Slightly curved panels create sufficient space for plant pots. In future, the plants will overgrow the façades. Diverse factors and requirements for each façade resulted in a “flexible second skin” concept for the elevations’ new look. Such skin integrates all necessary elements, which improve technical conditions and harmonize with neighboring buildings and green areas. The design of a coherent shell was followed by modifications inside the building. The legibility and efficiency of the layout was enhanced by minor spatial rearrangements (new staircase, student work, and recreation areas), introduction of a green new atrium, and use of color. Damaged materials were pointed out for replacement, adding to coherency of new design with emphasis on the durability and cost issues. Current questionable aesthetics and low-quality materials were replaced to unify multi-material interior fit-out. The use of simple forms and low-key colors improves the aesthetics and legibility of the buildings’ interiors. Lighting system is optimized to provide energy savings and to improve the existing learning and working conditions. Surface-mounted, in-built, spot, and linear luminaries as well as technical lights with extra protection are introduced (e.g., Zumtobel light fittings). Pro-environmental, low-emission, healthy, non-toxic, reused, and recyclable materials were chosen for interior finishing. Such materials with the Cradle-to-Cradle certificate as suspended ceilings Armstrong Perla OP0, 9, AEG glass and Strava carpets were chosen. Only certificated, locally sourced wood was used. Furthermore, the whole design process was designed in line with the rules of 3R (Reduce-Reuse-Recycle) (Petzet 2012) concept: modifications are limited to necessary renovation works, the original structure of the building remains untouched, and new elements are created rationally to last and to save energy and resources.

**Fig. 10:**
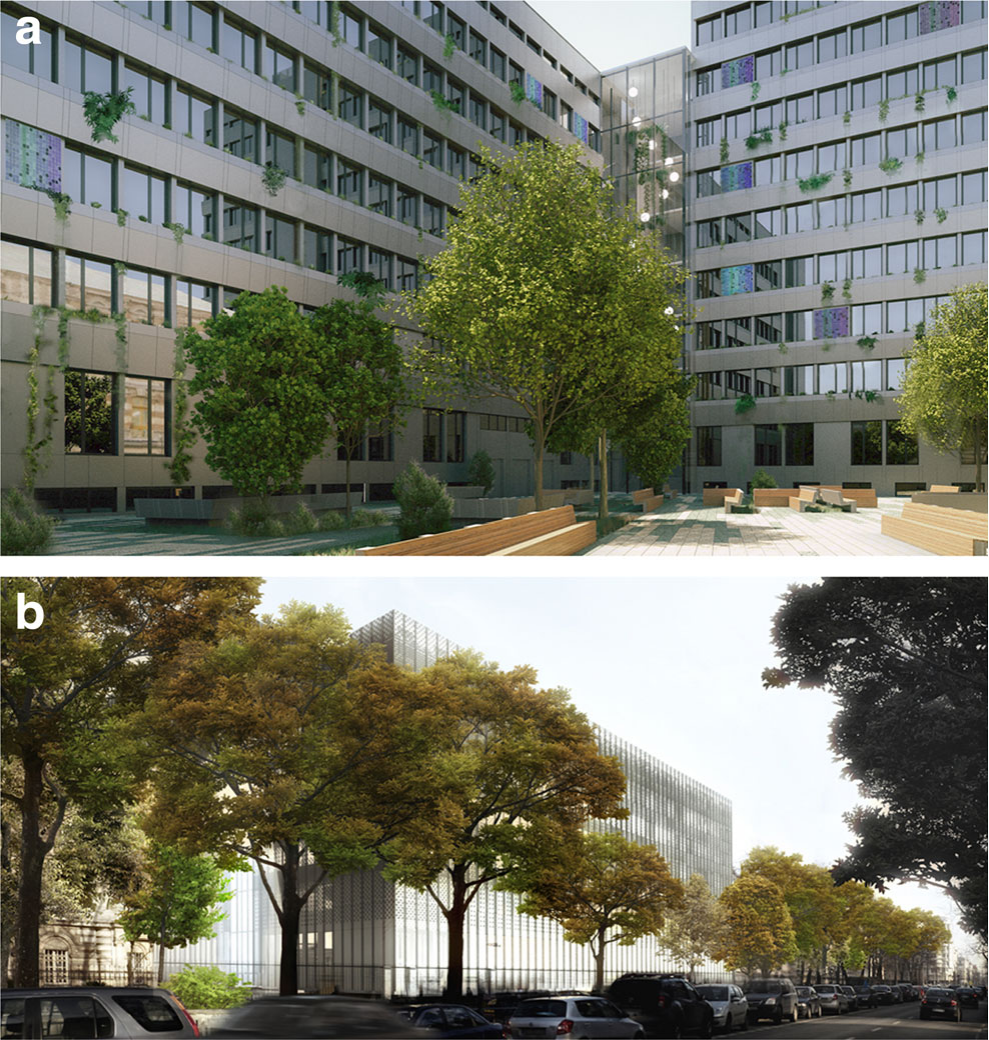
Faculty of Building Services, Hydro and Environmental Engineering—proposition of the “new skin” screening mechanical areas. BP phase (KODnZEB 2015–2017)

Existing courtyard, green areas, and urban infrastructure is redesigned to create a modern, multipurpose, and lively public space. The renovation of space around FBSHEE requires a complex approach as it is a part of main WUT campus. Courtyard is presently mainly used as a random parking place. In future, it will be transformed to a lively public square with wooden furniture, eco flooring, and other organic green arrangements. The façades of the modernized building around the plaza will be overgrown (an inbuilt façade green system has been described earlier). The external planted areas are linked with building arrangement through a new glass atrium. This linkage opens the Faculty building to a new plaza and historic surroundings. Moreover, the atrium connects the external green areas with the new internal solutions: a multi-storey green wall in the atrium, plants in concrete pots on the balconies, and other small green forms. They enhance the building’s common space natural environment and create comfortable and healthy areas for work and rest, with high ventilation parameters and good daylight access. The introduction of plants inside the building contributes to the acoustic comfort, absorbs indoor pollutants (VOC), and improves the air quality (Montacchini et al. 2017; Luz 2011). Moreover, it has beneficial effects on users’ psychological well-being: it reduces stress and positively affects emotions and concentration (Montacchini et al. 2017). The Biophilia I and II from the WELL Certification concept was followed in order to enhance the site. Thus, the renovation of the FBSHEE is a more complex process than energy efficiency measures and aims to create a functional and healthy work environment in an nZEB standard (Fig. 10).

## Executed nZEB retrofit—comparison of performance

During the grant’s development, Powerhouse Kjorbo, an office building from the 1980s, was chosen as a Norway reference case. This building was redesigned in 2014 by Snohetta and executed in collaboration with Skanska, Entra Eiendom (developer), ZERO (environmental organization), Asplan Viak, and Sapa. This pilot project, led by the Norwegian Research Centre on Zero Emission Buildings, is located in Sandvika Business Park, which consists of nine buildings. Two of them (total heating floor area of 5.180 m^2^) were chosen for retrofitting. Before the modernization, their average energy use was estimated at 240 kWh/m^2^/a. Due to unsealed windows, installations, and existing thermal bridges, heat losses were high. Glass and aluminum facades without adequate shading elements resulted in poor indoor conditions (Sørensen et al. 2017). The project of modernization was developed with the use of BIM software (for 3D inventory, existing greenery, and installation systems) and laser scan (for evaluation of existing load-bearing structure). New installation systems, highly insulated walls, windows with good insulation performance, and limited thermal bridges resulted in a good thermal performance of the building’s envelope (achieved air-tightness parameter is 0.23 air exchanges per hour with pressure of 50 Pa). The building produces more energy than it consumes. After redesigning, the total energy demand was reduced by 80% and it uses 80% of usable energy needed by a standard (Norwegian) building with energy class C (Klimowicz 2018). Moreover, photovoltaic modules placed on the rooftop produce more energy for electricity (221 MWh/year during the first year and 232 MWh during the second) than the building requires. In comparison, ventilation, lighting, heating, and cooling require 145,000 kWh/year. Ventilation pre-heating, cooling, and water heating in the building are run on geothermal energy (two heat pumps taking energy from ten 200-m deep boreholes). Lighting, fans, and materials are optimized to reduce energy consumption. The excess of heat from the server room is recuperated. The radiators are working only during the cold months. Powerhouse uses also solar energy generated by the photovoltaic modules located on the roof of the building and on the roof on its parking lot (1556 m^2^). This system provides the electricity for the building and gives the surplus to the city grid. The modernization of Powerhouse Kjorbo resulted in lower annual energy use (25.1 kWh/m^2^) and surplus of produced energy (when photovoltaic system is used) (Table 3).

**Table 3:** FBSHEE and Powerhouse Kjorbo—comparison of performance

Parameter	FBSHEE	Powerhouse Kjorbo
Total heated area (m2)	17,476.95 m^2^	5180 m^2^
Annual energy use before modernization	144 kWh/m^2^/a	240 kWh/m^2^/a
After modernization		
Annual energy use	17.6 kWh/m^2^/a^a^	25.1 kWh/m^2^
Annual energy production	11.2 kWh/m^2^/a^a^	44.1 kWh/m^2^
Annual energy from PV modules	57 MWH/year^a^ (3.0 kWh/m^2^/a^a^)	221 MWh/year
Annual energy required for ventilation, lighting, heating, and cooling	330,825 kWh/year^a^	145,000 kWh/year

^a^Predicted indicator based on simulation results for FBHSEE

^b^Values for Kjorbo building were measured during a 2-year use of the building

The project of modernization aimed also to achieve lower carbon emissions. Thus, pro-environmental, durable, and reused materials were used. The choice of materials (e.g., timber cladding, reused glass and concrete, recycled plastic bottles transformed to vertical baffles) reduced carbon emission by 70% in comparison with standard building. Thus, a holistic project of Powerhouse Kjobro complies with Norwegian norms (produced energy compensates energy used during building’s energy life cycle) and exceeds the nearly zero-energy standard but—to some extent—can be instructive for KODnZEB project in Warsaw (Table 1, Table 2).

In both buildings, as shown in Table 4, modern technologies were used such as demand control ventilation with heat recovery, led lighting system with individual control, and PV panels. However, without proper analysis of the building’s structure and its use, the application of these technologies would not bring the expected effect. In both cases, the technologies have been adapted to the carefully designed architectural and construction project and also to the individual character of the building.

**Table 4:** FBSHEE and Powerhouse Kjorbo—comparison of characteristic parameters after renovation

Parameter	FBSHEE	Powerhouse Kjorbo
U-values		
External wall	0.2	0.13
Roof	0.15	0.08
Floor on ground	0.25	0.14
Windows	0.90	0.80
Systems		
Heating system	Source: CHP, district heating, and heat pump installation with water radiators	Source: geothermal heat pump in addition to waste heat from the data/server room will cover the heating and cooling demand. Installation: radiators located in the central part of the building
Ventilation system	Auditoriums: decentralized mechanical supply and exhaust ventilation system, demand controlled ventilation, air supply from the double façade space Office rooms: decentralized mechanical supply and exhaust ventilation system, air supply from the double façade space, common exhaust duct Staircase: mechanical ventilation with heat recovery (efficiency 85%)	Demand controlled ventilation, minimized length of supply and exhaust air ducts; some structural elements of the building were used (for example, air exhaust provided by the staircase); displacement air distribution system, heat recovery efficiency 85%, openable windows
Cooling system	Source: heat pump—free cooling during the summer Cooling system only in office rooms	Source: heat pumps—free cooling during the summer
Hot water system	Source: CHP, district heating, and heat pump	Source: geothermal heat pump
Lighting system	LED lighting sources, individual lighting control	LED lighting sources, individual lighting control for each work space (about 15 m^2^).
Energy production	PV panels, combined heat, and power production in CHP units	PV panels

## Conclusions

Management of the interdisciplinary design process, as well as interaction between Polish and Norwegian designers which included transfer of know-how, proves that the main issue is establishment of a clear set of sustainable issues defined at the early design stages. Such procedure allows achievement of a strong synergic effect. This approach does not limit architectural creativity in any way, forming an environment with high health and well-being user capacity. This project is the final result of numerous analyses and calculations conducted by environmental engineers, electricians, and historic and landscape consultants.

It should be noted that the history of a potential construction success starts very early. LCA and energy data should be provided already within the feasibility study. Analysis showing potential effects of chosen design solutions also should be prepared at that stage. When leading a multidisciplinary design process, members of the whole team concentrate on uniform understanding of the aims and tasks defined for each phase of the design. Achieved standard depends on the design quality of each of the technical solutions merged together into a uniform synergic whole.

Decision-making by all designers is consciously made and with awareness that a minor change in one discipline may cause a major change in other ones. This attitude can be perceived as additional added value. Energy simulations are prepared already at the design concept stage, whereas the leading designer is responsible for the modeling of final solutions aiming at assumed parameters.

One of the main tasks which we still are facing is giving the building sector a better knowledge—wider education scoping also environmental solutions and understanding the need to a holistic approach to design process and implementation of environmentally friendly solutions. The assessment and all information contained within this paper was extracted from the research contained provided during the grant.

As researchers and active professionals, we assumed that our proposition should show that the nZEB energy benchmarks accepted in Poland can be moved to a higher standard. We hope that this case study will be an inspiration for further improvement of energy parameters and acceptance that use of alternative energy sources can be provided to the benefit of all interested parties.

## References

[CR1] (2008) Communication from the Commission to the European Parliament, The Council, The European Economic and Social Committee and the Committee of Regions Public Procurement for better environment dated

[CR2] (2010) Making our cities attractive and sustainable. How the EU contributes to improving the urban environment Luxembourg: Publications Office of the European Union

[CR3] Aelenei L, Petran H, Tarrés J, Riva G, Ferreira A, Camelo S, Corrado V, Šijanec-Zavrl M, Stegnar G, Gonçalves H (2015). New challenge of the public buildings: nZEB findings from IEE RePublic_ZEB Project. Energy Procedia.

[CR4] Beccali M, Galatioto A, Leone G, Longo S (2013). Is the NZEB benchmarking approach suitable for assessing energy retrofit design?. Appl Mech Mater.

[CR5] Becchio C, Bottero M, Corgnati S, Ghiglione C (2015). nZEB design: challenging between energy and economic targets. Energy Procedia.

[CR6] Brambilla A, Salvalai G, Imperadori M, Sesana MM (2018). Nearly zero energy building renovation: from energy efficiency to environmental efficiency, a pilot case study. Energy and Buildings.

[CR7] Chludzińska M, Bogdan A (2017). The role of the front pattern shape in modelling personalized airflow and its capacity to affect human thermal comfort. Build Environ.

[CR8] Christoforidis G, Melandri D, Branciforti V, Nousdilis A, Papagiannis G, Penalvo E (2016) Meeting of energy skills: an educational framework for energy professionals in NZEB. EEEIC 2016 - International Conference on Environment and Electrical Engineering (2016) Published by Institute of Electrical and Electronics Engineers Inc.

[CR9] Commission Delegated Regulation (EU) no 244/2013 supplementing Directive 2010/31/EU of the European Parliament and of the Council on the energy performance of buildings by establishing a comparative methodology framework for calculating cost-optimal levels of minimum energy performance requirements for buildings and building elements

[CR10] Cuce E (2017). Thermal regulation impact of green walls: an experimental and numerical investigation. Appl Energy.

[CR11] Dalla Mora T, Cappelletti F, Peron F, Romagnoni P, Bauman F (2015). Retrofit of an historical building toward NZEB. Energy Procedia.

[CR12] Dalla Mora T, Cappelletti F, Peron F, Romagnoni P, Bauman F (2015). Towards nZEB buildings: a historical building case study. REHVA European HVAC Journal.

[CR13] Ruocco di G, Sicignano C, Sessa A (2016) Integrated methodologies energy efficiency of historic buildings. International High-Performance Built Environment Conference – A Sustainable Built Environment Conference 2016 Series (SBE16), IHBE 2016

[CR14] EPBD recast 2010/31/UE dayed 15.05.2010 concerning building energy characteristic

[CR15] Fernández-Cañero R, Urrestarazu LP, Salas AF (2012). Assessment of the cooling potential of an indoor living wall using different substrates in a warm climate. Indoor Built Environ.

[CR16] Fouche M, Crawford RH (2016) Towards integrated approach for evaluating both the life cycle environmental and financial performance of a building. A review. International High-Performance Built Environment Conference – A Sustainable Built Environment Conference 2016 Series (SBE16), IHBE 2016

[CR17] Hamdy M, Hasan A, Siren K (2013). A multi-stage optimization method for cost-optimal and nearly-zero-energy building solutions in line with the EPBD-recast 2010. Energy and Buildings.

[CR18] Harkoussa F, Fardouna F, Biwolec PH (2018). Multi-objective optimization methodology for net zero energy buildings. Journal of Building Engineering.

[CR19] Integrated Design (2012–2014) Official project website www.integrateddesign.eu

[CR20] International High-Performance Built Environment Conference – A Sustainable Built Environment Conference 2016 Series (SBE16), IHBE 2016

[CR21] Jagemar L, Schmidt M, Allard F, Heiselberg P, Kurnitski J (2011). Towards nZEB—some examples of national requirements and roadmaps. REHVA European HVAC Journal.

[CR22] Kang H (2015). Development of a decision support tool for nZEB (nearly zero emission building) at the early design stage. Journal of Asian Architecture and Building Engineering.

[CR23] Kantola M, Saari A (2016). Identifying and managing risks involved in the transition to the EU nZEB decree. Facilities.

[CR24] Kantola M, Saari A (2016). Project delivery systems for nZEB projects. Facilities.

[CR25] Karima M, Altan H (2016) Interactive building environments: a case study university building in UAE

[CR26] Karlessi T, Kampelis N, Kolokotsa D, Santamouris M, Standardi L, Isidori D, Cristalli C (2017). The concept of smart and NZEB buildings and the integrated design approach. Procedia Engineering.

[CR27] Klimowicz J (2018) Chosen case studies of nZeb retrofit buildings. In: Rynska E, Kozminska U, Rucinska J, Zinowiec-Cieplik K, Szybinska-Matusiak B (eds) Design solutions for nZeb retrofit buildings. IgI Global, p 209–227

[CR28] Kurnitski J, Allard F, Braham D, Goeders G, Heiselberg P, Jagemar L, Kosonen R, Lebrun J, Mazzarella L, Railio J (2012) How to define nearly net zero energy buildings nZEB. REHVA Journal (2011):6–12

[CR29] Kurnitski J, Feldmann C, Heiselberg P, Massarella L, Sartori I, Voss K, Wahlström Å (2013) Present energy performance requirements and nZEB targets in some selected countries. Cost optimal and nearly zero-energy buildings (nZEB): definitions, calculation principles and case studies. pp 31–46

[CR30] Kurnitski J, Buso T, Corgnati SP, Derjanecz A, Litiu A (2014). nZEB definitions in Europe. REHVA European HVAC Journal.

[CR31] Kwiatkowski J (2017) “Ekonomiczne aspekty modernizacji budynków użyteczności publicznej do standard nZEB”. In: Sowa J (ed) 2017: Budynki o niemal zerowym zużyciu energii. Oficyna Wydawnicza PW

[CR32] Kwiatkowski J, Mijakowski M, Trząski A (2017) The measures for achieving nZEB standard of retrofitted educational building for specific polish location – case study. 10.1051/e3sconf/20172200098

[CR33] Lindkvist C, Karlsson A, Sørnes K, Wyckmans A (2014). Barriers and challenges in nZEB projects in Sweden and Norway. Energy Procedia.

[CR34] Ling T, Chang Y (2018). Well-being, health and urban coherence-advancing vertical greening approach toward resilience: a design practice consideration. J Clean Prod.

[CR35] Liu Sh, Song D, Yu B (2016) The objective and methodology of urban climate map for the city of Xiamen. International High-Performance Built Environment Conference – A Sustainable Built Environment Conference 2016 Series (SBE16), IHBE 2016

[CR36] Lohr VI, Relf D (1992). The contribution of interior plants to relative humidity in an office. The role of horticulture in human well-being and social development.

[CR37] Luz C (2011) Planting healthier indoor air. Environ Health Perspect 119(10) 10.1289/ehp.119-a426 PMC3230460 22069776

[CR38] Marszal AJ, Heiselberga P, Bourrelle JS, Musall E, Voss K, Sartori I, Napolitano A (2011). Zero energy building—a review of definitions and calculation methodologies. Energy and Buildings.

[CR39] Mijakowski M, Rucińska J, Sowa J, Narowski P (2017) Koncepcja poprawy środowiska wewnętrznego w przykładowym budynku użyteczności publicznej modernizowanym do standardu nZEB, Building Physics in Theory and Practise

[CR40] Montacchini E, Tedesco S, Rondinone T (2017). Greenery for a university campus: does it affect indoor environmental quality and user well-being?. Energy Procedia.

[CR41] Ó’Riain M, Harrison J (2016). Cost-optimal passive versus active nZEB. How cost-optimal calculations for retrofit may change nZEB best practice in Ireland. Archit Sci Rev.

[CR42] Perini K, Bazzocchi F, Croci L, Maglioccoa A, Cattaneoc E (2017). The use of vertical greening systems to reduce the energy demand for air conditioning. Field monitoring in Mediterranean climate. Energy and Buildings.

[CR43] Petzet M (2012) Reduce, reuse, recycle. Hatje Cantz Verlag

[CR44] Pomfret L, Hashemi A (2017). Thermal comfort in zero energy buildings. Energy Procedia.

[CR45] Raji B, Tenpierik MJ, van den Dobbelsteen A (2015). The impact of greening systems on building energy performance: a literature review. Renew Sust Energ Rev.

[CR46] Rynska ED (2008) Rehabilitation and adaptive reuse of historic buildings in Poland, chapter in “Harmonization between Architecture and Nature. Eco-Architecture II”. WIT Press

[CR47] Rynska ED (2018) 3XE: efficiency, ecosphere, economics, chapter in “Design solutions for nZEB retrofit buildings”. IGI Global Disseminator of Knowledge

[CR48] Rynska ED, Bartkiewicz P (2017) “Zintegrowane projektowanie budynku, rozruch i odbiory techniczne instalacji”. In: Sowa J (ed) 2017: Budynki o niemal zerowym zużyciu energii. Oficyna Wydawnicza PW

[CR49] Rynska ED, Kozminska U (2018) Existing buildings: how to meet an nZeb standard—the Architect’s perspective chapter in “Design solutions for nZEB retrofit buildings”. IGI Global Disseminator of Knowledge

[CR50] Singh B, Roy P, Spiess T, Venkatesh B (2015) Sustainable integrated urban & energy planning, the evolving electrical grid and urban energy transition. The Centre for Urban Energy. Ryerson University

[CR51] Sørensen AL, Andersen I, Walnum HT, Justo-Alonso M, Fufa SM, Jenssen B, Radstoga O, Hegli T, Fjeldheim H (2017) Pilot Powerhouse Kjobro. As Built Report, Sintef

[CR52] Sowa J (ed) (2017) Budynki o niemal zerowym zużyciu energii. Oficyna Wydawnicza PW

[CR53] Szalaya Z, Zöldb A (2014). Definition of nearly zero-energy building requirements based on a large building sample. Energy Policy.

[CR54] Uribe O, Santos M, Garcia-Alegre M, Guinea D (2015) A context-awareness architecture for managing thermal energy in an nZEB building. 2015 IEEE 1st International Smart Cities Conference, ISC2 2015 (2015) Published by Institute of Electrical and Electronics Engineers Inc.

[CR55] Wagner A (2015). Czas I miejsce.

[CR56] Zeiler W (2010) Integral design of NZEB a perquisite for 0 impact buildings. Proceedings SB10 Euregion: Towards 0-impact buildings and environments held in October 2010 in Maastricht, Liege and Aachen (2010) Published by in-house publishing

[CR57] PN-82/B-02403 Ogrzewnictwo Temperatury obliczeniowe zewnętrzne (Heating. Calculation of internal temperatures)

[CR58] PN-EN 15251 Kryteria środowiska wewnętrznego, obejmujące warunki cieplne, jakość powietrza wewnętrznego, oświetlenie i hałas (Internal standard criteria concerning heating standards, internal air quality, light systems and noise)

[CR59] A national plan with the aim to achieve a higher number if buildings possessing efficient energy standards, introduced by the Chairman of the Council of Ministers 22.06.2015

[CR60] Resolution of the Minister of Infrastructure concerning technical parameters to be achieved by buildings and their location dates 12 April 2012 (Legal Act no 75, pos. 690 with later amendments)

[CR61] DesignBuilder wersja 4.2.0.054. http://www.designbuilder.co.uk

[CR62] Krajowy Ośrodek Bilansowania i Zarządzania Emisjami official website www.kobize.pl

